# Nonstructural protein 4 of human norovirus self-assembles into various membrane-bridging multimers

**DOI:** 10.1016/j.jbc.2024.107724

**Published:** 2024-08-28

**Authors:** Adrien Royet, Rémi Ruedas, Laetitia Gargowitsch, Virginie Gervais, Johann Habersetzer, Laura Pieri, Malika Ouldali, Maïté Paternostre, Ilse Hofmann, Thibault Tubiana, Sonia Fieulaine, Stéphane Bressanelli

**Affiliations:** 1 Université Paris-Saclay, CEA, CNRS - Institute for Integrative Biology of the Cell (I2BC), Gif-sur-Yvette, France; 2 Sanofi, Integrated Drug Discovery, Vitry-sur-Seine, France; 3 Université Paris-Saclay, CNRS, Laboratoire de Physique des Solides, Orsay, France; 4 Core Facility Antibodies, German Cancer Research Center, Heidelberg, Germany

**Keywords:** plus-stranded RNA virus, viral replication, membrane, viral protein, liposome, cryo-EM, lipid–protein interaction, protein assembly, nonstructural protein, norovirus

## Abstract

Single-stranded, positive-sense RNA ((+)RNA) viruses replicate their genomes in virus-induced intracellular membrane compartments. (+)RNA viruses dedicate a significant part of their small genomes (a few thousands to a few tens of thousands of bases) to the generation of these compartments by encoding membrane-interacting proteins and/or protein domains. Noroviruses are a very diverse genus of (+)RNA viruses including human and animal pathogens. Human noroviruses are the major cause of acute gastroenteritis worldwide, with genogroup II genotype 4 (GII.4) noroviruses accounting for the vast majority of infections. Three viral proteins encoded in the N terminus of the viral replication polyprotein direct intracellular membrane rearrangements associated with norovirus replication. Of these three, nonstructural protein 4 (NS4) seems to be the most important, although its exact functions in replication organelle formation are unknown. Here, we produce, purify, and characterize GII.4 NS4. AlphaFold modeling combined with experimental data refines and corrects our previous crude structural model of NS4. Using simple artificial liposomes, we report an extensive characterization of the membrane properties of NS4. We find that NS4 self-assembles and thereby bridges liposomes together. Cryo-EM, NMR, and membrane flotation show formation of several distinct NS4 assemblies, at least two of them bridging pairs of membranes together in different fashions. Noroviruses belong to (+)RNA viruses whose replication compartment is extruded from the target endomembrane and generates double-membrane vesicles. Our data establish that the 21-kDa GII.4 human norovirus NS4 can, in the absence of any other factor, recapitulate *in tubo* several features, including membrane apposition, that occur in such processes.

Single-stranded, positive-sense RNA ((+)RNA) viruses are a large class of viruses, including several major human pathogens such as coronaviruses (*e.g.*, SARS-CoV-2), flaviviruses (*e.g.*, dengue virus and Zika virus) or hepatitis C virus (HCV). One striking feature of (+)RNA viruses is their capacity to build their replication complex on the cytosolic side of remodeled host endomembranes, providing a safe environment to replicate the viral genome prior to encapsidation in new viral particles (1, 2). The processes of virus-induced membrane remodeling are poorly understood, but the ultrastructural features of membranous replication compartments have led to a division in two "morphotypes": (+)RNA viruses replicating their genomes in single-membrane, invaginated vesicles, such as dengue virus, Zika virus, and other flaviviruses; and (+)RNA viruses replicating their genomes in double membrane vesicles (DMVs) extruded from the target endomembrane, the prototype of which is HCV (3). Other major pathogenic (+)RNA viruses where replication is associated with generation of DMV are coronaviruses, picornaviruses, and noroviruses, although there seem to be differences in the relevance of DMV to different (+)RNA viruses. Thus DMV are clearly the sites of coronavirus replication (4, 5), but single-membrane vesicles (SMVs) that appear in greater abundance during replication are likely the sites of picornavirus (6) and norovirus (7, 8) replication.

Noroviruses are members of Caliciviridae, a family of small nonenveloped (+)RNA viruses causing a broad spectrum of diseases in humans and animals (9). Human noroviruses are the main cause of epidemic nonbacterial gastroenteritis worldwide. Among Caliciviridae, noroviruses are themselves genetically diverse and are classified into some ten genogroups GI to GX, each genogroup comprising several genotypes (10). Genogroups I, II, IV, VIII, IX infect humans, while genogroup V infects mice. Genogroup II genotype 4 noroviruses (GII.4) have been the main circulating human noroviruses for more than 20 years (10, 11). For instance, three distinct GII.4 strains were associated with gastroenteritis outbreaks in Den Haag (2006b), New Orleans (NO, 2009), and Sydney (2012).

Norovirus intracellular replication involves translation of the genomic RNA into a single replication polyprotein ORF1 comprising six proteins NS1-2, NS3, NS4, NS5, NS6, and NS7. Functions for the last three proteins are clear: NS6 is the viral protease that processes the polyprotein into the six end-products. NS7 is the RNA-dependent RNA polymerase that uses first the genomic RNA as a template to synthesize negative-sense RNAs, likely by a *de novo* (without primer) mechanism (12). NS7 then uses NS5 as a protein primer for positive-sense RNA synthesis, both of new genomic RNAs and of subgenomic RNAs from which the structural proteins VP1 and VP2 are synthesized. NS5 (also called VPg for genome-linked viral protein) is thus covalently linked to the genomic and subgenomic RNAs and is an essential factor for their translation. The first three proteins in ORF1 (NS1-2, NS3, and NS4) are less well characterized, but it has grown clear that they are the drivers of endomembrane remodeling for noroviruses (8, 13). Expression of human ORF1 in human cells induces vesicular membrane alterations built from endoplasmic reticulum (ER), including SMVs, DMVs, and multimembrane vesicles (8). When expressed individually, NS1-2, NS3, and NS4 also induce vesicular membrane alterations from ER: NS1-2 induces protrusions forming long tubular structures, NS3 induces the formation of convoluted membranes, and NS4 induces the formation of both SMVs, DMVs, and convoluted membranes (8, 14). Thus, while all three of NS1-2, NS3, and NS4 nonstructural proteins contribute to membrane alterations, NS4 appears to be the most important remodeling protein, being able to reproduce virus-induced intracellular membrane ultrastructures by itself (8). Nevertheless, little is known about its structure, its function and, most importantly, how it interacts with and deforms membranes. Thus, in order to better understand the role of human norovirus NS4 during the viral lifecycle, we conducted a biochemical and structural study aiming at characterizing human NoV NS4. In the present work, we thus present data on a recombinant GII.4 NS4 that clarifies its functions in endomembrane rearrangements by noroviruses.

## Results

### Production and purification of GII.4 NS4 and generation of antibodies

**Figure 1:**
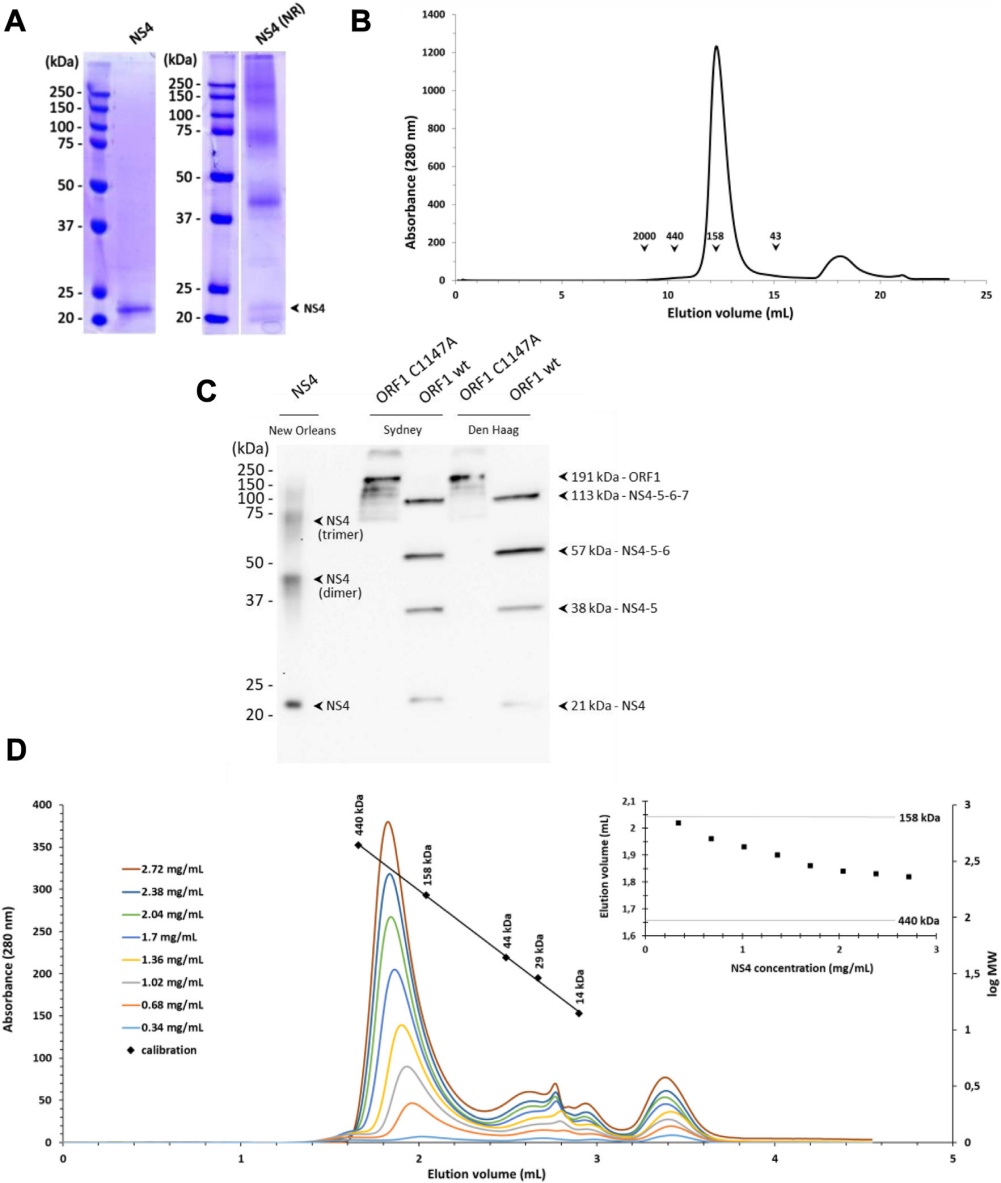
**Biochemical analysis of purified recombinant NS4.***A*, 6His-tagged NS4 protein from GII.4 New Orleans variant was produced in bacteria and purified in presence of DDM. Protein sample was analyzed by SDS-PAGE in reducing (*left*) and nonreducing (NR, *right*) conditions. The *arrow* indicates the band corresponding to the expected molecular weight of denatured monomeric NS4. *B*, the elution profile of DDM-solubilized NS4 was analyzed by size-exclusion chromatography on a Superdex 200 10/300 GL column, and compared to standard globular molecular weight markers whose elution volumes are indicated. *C*, WT or C1147A mutated ORF1 from GII.4 Sydney or Den Haag variants were expressed by a wheat-germ cell-free expression system. Expression profiles were analyzed by Western blot and compared to pure 6His-tagged New Orleans NS4. Since we used anti-NS4 antibody, only fragments containing NS4 are detected. *Arrows* indicate the bands corresponding to uncleaved full-length C1147A ORF1 or processing intermediates generated after self-cleavage of WT ORF1. The expected sizes of the proteins were calculated by taking into account the presence of a Strep-tag at both N- and C-terminal extremities (15). *D*, elution profile of DDM-solubilized NS4 at different concentrations was analyzed on an analytical Superdex 200 5/150 column. NS4 samples at constant volume but decreasing concentrations were injected onto the column and resulting chromatograms were superimposed for comparison. The calibration curve of the column using molecular weight markers is indicated. The inset represents the elution volume of the main peak as a function of the input protein concentration. The elution volumes of 158 and 440 kDa molecular weight markers are indicated with a *thin line*. DDM, n-dodecyl-β-D-maltoside; GII.4, genogroup II genotype 4; NS, nonstructural.

We expressed in *Escherichia coli* an N terminally 6-histidine tagged version of NS4 from genotype GII.4 (an isolate of NO 2009 variant, 179 residues not counting the tag, *cf.* Experimental procedures and sequence in Fig. S1). We found that after cell lysis, detergent above the critical micellar concentration was required to keep NS4 soluble. We purified NS4 by successive affinity chromatography steps (Fig. S2), with 0.04% n-dodecyl-β-D-maltoside (DDM) in the final buffer. We typically obtained 10 mg of pure NS4 from 5 l of cell culture. The purified protein migrates close to its expected molecular mass of 21 kDa in SDS-PAGE (Fig. 1*A*, left). Mass spectrometry after trypsin digestion confirms the identity of recombinant NS4 with 97% sequence coverage (not shown). NO NS4 elutes within a single peak from a size-exclusion chromatography column (Fig. S2*D*, SDS-PAGE). The NS4 elution volume is close to the 158 kDa molecular mass marker (Fig. 1*B*), suggesting oligomerization and/or association with a DDM detergent micelle. Remarkably, simply using nonreducing conditions in SDS-PAGE yields a ladder of at least five bands (Fig. 1*A*, right), which we interpret as crosslinking between NS4 molecules by several of the five cysteines in NO NS4.

This recombinant NS4 was used to immunize mice and obtain specific anti-NS4 mAbs. Sensitive detection of NO NS4 by Western blots using one of the mAbs detects the expected 21-kDa band, but also several additional upper bands matching the lower part of the ladder seen in nonreducing conditions (Fig. 1*C*, lane NS4). This confirms that the higher molecular weight bands observed in nonreducing SDS-PAGE correspond to NS4, and suggests that the apparent “dimer” and “trimer” bands are partially resistant to SDS. The same mAb also recognizes NS4 when produced in the context of the norovirus polyprotein from two other GII.4 strains (Sydney 2012 and Den Haag 2006b), despite 10 to 20 residue substitutions compared to NO NS4 (Fig. S1). Indeed, the antibody detects the full-length uncleavable ORF1 expressed by *in vitro* translation using a wheat germ extract (we introduced in ORF1 the C1147A mutation to inactivate the NS6 protease activity, see reference (15)) (Fig. 1*C*, lanes ORF1 C1147A). The antibody also detects NS4-containing processing intermediates when WT ORF1 is expressed (Fig. 1*C*, lanes ORF1 wt), because active NS6 protease spontaneously processes ORF1 in the *in vitro* translation mixture as we previously observed (15). Of note, cleavage fragments identified by the Western blot are not the same as in our previous work because we then used anti-Streptag antibodies that recognize the Strep-tags inserted at both N and C termini of ORF1 (15). The bands identified using the anti-NS4 antibody (this work) validate that this *in vitro* system recapitulates the well described NoV ORF1 proteolytic maturation process, with transiently stable NS4-containing intermediates after cleavage of the N-terminal NS1-2 and NS3 (16, 17).

We next sought to characterize the putative NS4 oligomers using an analytical size-exclusion chromatography approach. We therefore performed a systematic analysis of NS4 apparent molecular mass as a function of its input concentration. To do this, we injected on the column a constant volume of NS4 samples at decreasing concentrations, with protein diluted in the elution buffer containing a fixed 0.04% DDM. Comparison with molecular weight markers shows that on this smaller volume column the apparent molecular mass of NS4 lies between the 158 kDa marker and the 440 kDa marker and drops from midway between the two markers toward the 158 kDa marker as its input concentration decreases (Fig. 1*D*). This suggests that NS4 oligomers or aggregates dissociate with greater dilution.

Altogether, these data indicate that recombinant NS4 in DDM forms labile oligomers that can be cross-linked by intermolecular disulfide bonds. We thus performed all subsequent experiments with strong reducing agents in all buffers.

### 3D modeling and NMR analysis of GII.4 NS4

**Figure 2:**
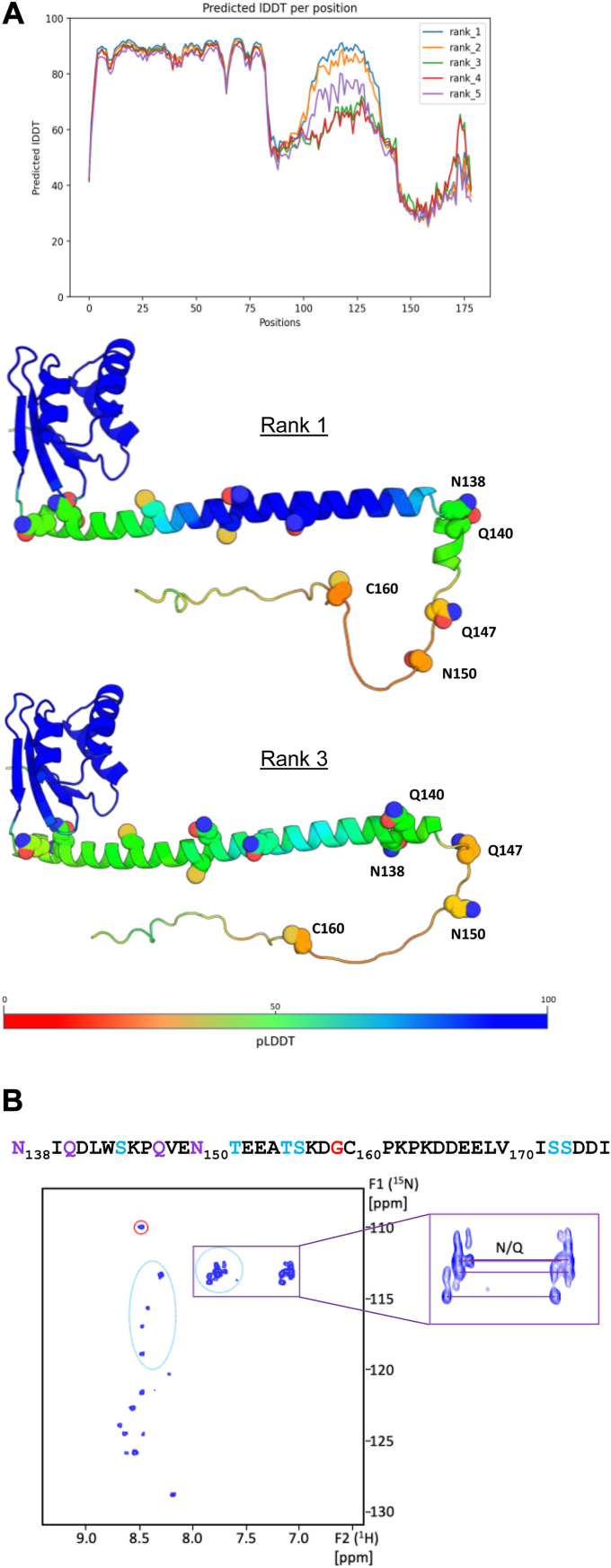
**Structural analysis of purified recombinant NS4.***A*, AlphaFold2 modeling of NS4 from the GII.4 New Orleans variant. *Top*, predicted LDDT score per residue for the five top-ranked models. *Bottom*, two models (ranks 1 and 3 *from top to bottom*) are represented as ribbons with alpha carbons colored according to the residue pLDDT score. The pLDDT scale is indicated, from the lowest (*red*, 0) to the highest confidence (*blue*, 100). Side chains for cysteines, asparagines, and glutamines in residues 86 to 179 are displayed as *spheres*. The most C-terminal cysteine (C160), asparagines (N138 and N150), and glutamines (Q140 and Q147) are labeled. *B*, 2D ^1^H-^15^N HSQC spectrum acquired with DDM-solubilized ^15^N-labeled NS4 is shown. The sequence corresponding to the ∼40-residue intrinsically disordered region is indicated, with Gly in *red*, Ser and Thr in *cyan*, and Asn and Gln in *purple*. The ^15^N-HN crosspeaks that could correspond to Gly/Ser/Thr residues are encircled on the spectrum using the same color code. Peaks connected by *thin lines* indicate possible correlations of Asn and Gln side chains -NH_2_ groups (zoomed in the inset). DDM, n-dodecyl-β-D-maltoside; GII.4, genogroup II genotype 4; HSQC, heteronuclear single quantum coherence; NS, nonstructural; pLDDT, predicted local-distance difference test.

We previously published a structural model, based on secondary structure predictions, which described NS4 as a ∼140-residue folded region ending with a long helical region of amphipathic nature, that we hypothesized would be involved in membrane interactions, and followed by a ∼40-residue intrinsically disordered region (8). With the advent of accurate atomic-level protein structure predictions (18), we next produced AlphaFold models of NO NS4 that allow a much finer look at NS4 structural organization (Figs. 2*A* and S1). There is an N-terminal globular domain (residues 3–85) predicted with high confidence (AlphaFold-predicted local-distance difference test (pLDDT) > 80) and made of a four-stranded beta-sheet apposed to three short connected helices. A search for structural homologs using DALI (19) returned no hit (Z-scores all below 4), indicating that there is no matching experimental structure in the Protein Data Bank. The globular domain is connected at various angles in various models to an amphipathic helical stretch of some fifty residues predicted with lower confidence (pLDDT∼60), except sometimes for a central portion (*e.g.*, model rank 1 in Fig. 2*A*). Finally, the C terminal ∼40 residues (N138-E179) are predicted with very low secondary structure content and pLDDT (<40), a hallmark of intrinsically disordered proteins (20), except around residue 175 where both may be higher (Figs. 2*A* and S1). Three cysteines are fully exposed in all models, one in the disordered region (C160) and two in the amphipathic helical stretch (Fig. S1), and are good candidates for the crosslinking observed in NO NS4 in noreducing conditions (Fig. 1*A*, right). Of the other two cysteines, one is fully buried in the globular domain and the other sits at its surface.

We further looked at NS4 by NMR using uniformly ^15^N-labeled protein. We acquired ^1^H-^15^N heteronuclear single quantum coherence (HSQC) spectra on a DDM solubilized NS4 sample (150 μM, 3.2 mg/ml) at different temperatures. We observed only few signals due to fast T2 relaxation, resulting in pronounced line broadening effects and a notable reduction of observable signals (data not shown), confirming that NS4 in DDM micelles and at higher concentrations is a large object. Increasing the temperature to 308 K led to an improved HSQC spectrum, resulting in the observation of approximately 20 backbone amide cross peaks (Fig. 2*B*). The poor spectral dispersion—especially in the ^1^H dimension—associated with these correlation peaks suggests a lack of structure associated with local flexibility, an inherent property of intrinsically disordered regions. Thus, of the ∼40 structurally disordered C-terminal amino acids predicted by AlphaFold, we could observe only half in the HSQC spectrum. These observable cross peaks are from residues with increased mobility (21). Even though the concentration and stability of the NS4 sample did not permit us to proceed with NMR assignment, we could extract valuable information from the analysis of the HSQC spectrum. The peak with the most upfield shifted ^15^N signal (109.0 ppm) is typically assigned to a glycine residue and likely corresponds to the single glycine residue present in the 40 C-terminal residues (G159, Figs. S1 and 2*B*). Side chains of asparagine and glutamine residues resonate in a shielded region compared to backbone amide protons, and several such NH_2_ correlations are visible in this region of the HSQC spectrum. The 40 C-terminal residues of NO NS4 contain two glutamine and two asparagine residues, but all of them precede G159 (N138, Q140, Q147, N150, displayed in Fig. 2*A*). Altogether, these observations suggest that the most disordered region of NS4 lies in the region spanning residues between N138 and G159, while its very C terminus would exhibit comparatively less mobility.

Thus, these NMR data refine and correct the AlphaFold model and delineate the most mobile part of NS4 as the region immediately downstream its central amphipathic helix.

### NS4 self-assembles onto synthetic membranes

**Figure 3:**
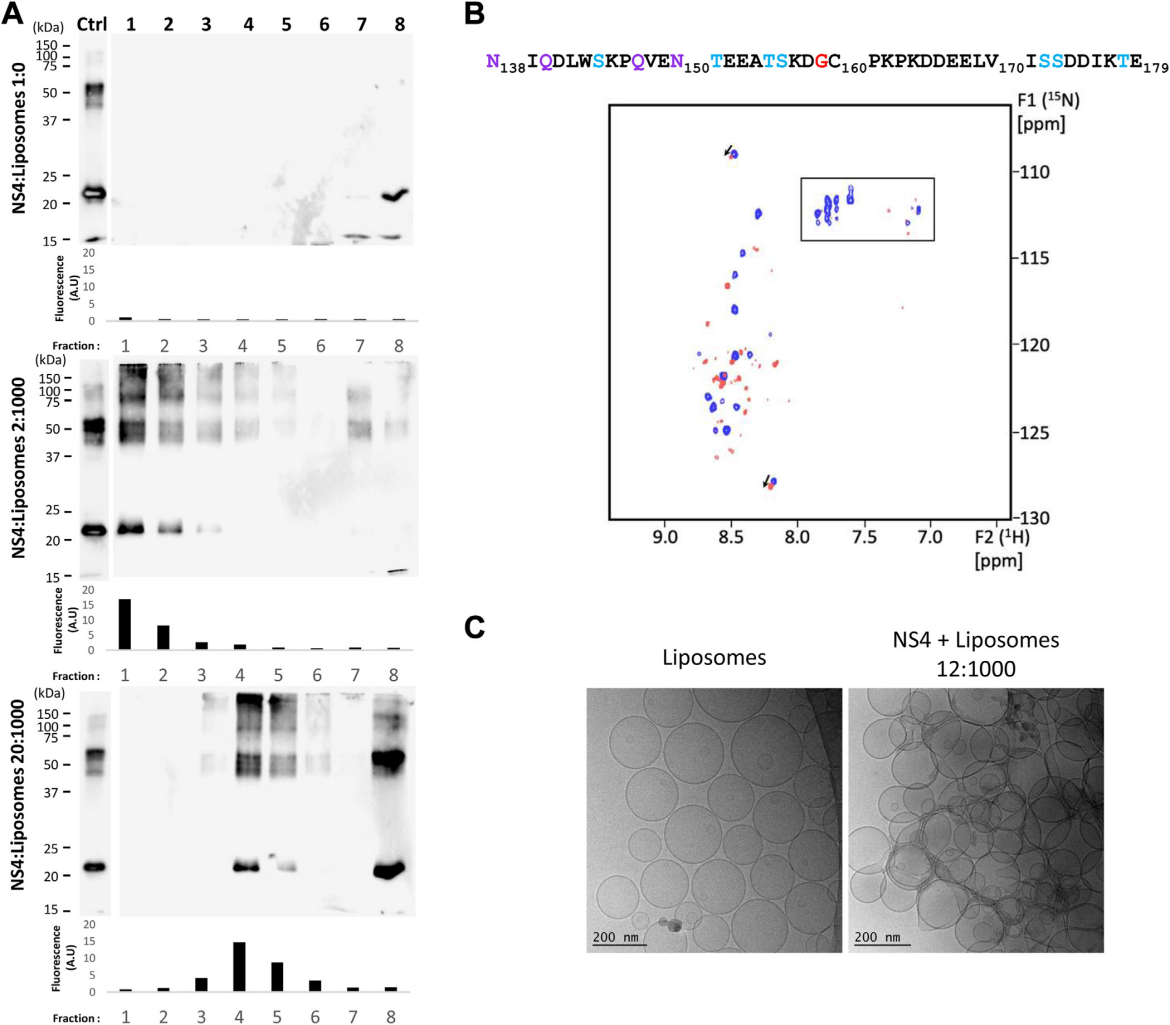
**Effects of NS4 interactions with liposomes.***A*, DDM-solubilized NS4 was incubated 10 min with PC-PS liposomes (ratios, 2:1000, 20:1000 NS4:lipids mol:mol) and subjected to a flotation assay on 0 to 20% sucrose gradients. After ultracentrifugation, 8 fractions were collected *from top to bottom* (lanes 1–8) and analyzed by Western blot using our anti-NS4 antibody. Control was NS4 without liposomes (ratio 1:0). One hundred nanograms of NS4 were loaded on each gel as an input control (lane Ctrl). In addition, the fluorescence of each fraction was measured to detect the location of fluorescent liposomes in the gradient; values are indicated below each corresponding Western blot membrane. *B*, 2D ^1^H-^15^N SOFAST-HMQC spectra acquired with DDM-solubilized NS4 incubated without (*blue*) and with liposomes (ratio 12:1000 NS4:lipids mol:mol) (*red*) are superimposed. Figure is annotated as in Fig. 2*B*. *C*, NMR sample of PC-PS liposomes incubated with DDM-solubilized NS4 was imaged by cryo-EM (*right panel*) and compared to liposomes without the protein (*left panel*). A representative region of each sample is shown. Scale bars are indicated. DDM, n-dodecyl-β-D-maltoside; NS, nonstructural; HMQC, hetero multiple quantum coherence; HSQC, heteronuclear single quantum coherence; PC, phosphatidylcholine; pLDDT, predicted local-distance difference test; PS, phosphatidylserine.

As NS4 is the main norovirus membrane altering protein in cells (see introduction), we next looked at its membrane interaction properties. To do that, we followed the general strategy of working with simple liposomes made of 89% egg phosphatidylcholine (PC) and 10% brain phosphatidylserine (PS), with 1% of the fluorescent dye Laurdan to label the membranes. First, we performed membrane flotation assays using a sucrose gradient (Fig. 3*A*). After ultracentrifugation and subsequent collection of eight density gradient fractions from top to bottom, NS4 in the absence of membranes was detected by Western blotting as expected only at the bottom of the gradient in fraction #8 (Fig. 3*A* top, NS4:liposomes 1:0). In contrast, when mixed with liposomes in a protein:lipids ratio of 2:1000 (mol:mol), most of NS4 was detected near the top of the gradient in the same fractions as the membranes (Fig. 3*A* middle). Strikingly, upon incubation with liposomes, NS4 also showed a migration toward higher molecular weight bands in SDS-PAGE. This indicates that NS4 not only interacts with membranes but also that this interaction induces the self-assembly of NS4 into SDS-resistant multimers. It is noteworthy that even the fraction of NS4 remaining at the bottom of the gradient tends to migrate to higher molecular weight bands in SDS-PAGE. This indicates that upon dilution into liposome suspensions, NS4 self-associates into membrane-bound but also membrane-independent multimers. It is unknown whether these membrane-independent multimers form because of an initial interaction with membranes and subsequent release or just because DDM partitions into the membranes, increasing the NS4:DDM ratio and the oligomerization state of NS4 as in Figure 1*D*. Increasing the protein:lipids ratio to 20:1000 (Fig. 3*A*, bottom) increased the amount of membrane-independent multimers but also showed comigration of NS4 with membranes lower in the gradient, indicating a higher density for the proteoliposomes and thus a higher amount of bound NS4.

To assess how the NS4 disordered region is affected in the context of liposome interaction, a SOFAST hetero multiple quantum coherence (HMQC) spectrum was recorded at 308 K for a ^15^N-NS4 sample in the presence of liposomes (at a final protein:lipids ratio of 12:1000 (mol:mol)) and compared with a SOFAST HMQC spectrum recorded without liposomes. Several notable changes were observed between the two spectra (Fig. 3*B*). A substantial decrease in the peak intensities was observed in the presence of the PC/PS liposomes, due to slower tumbling of the protein in the context of liposome interaction and/or protein self-association. Most of the NH_2_ correlations disappeared altogether in the Q/N region. The HN correlation peak of the presumed glycine did not disappear but became weaker and was furthermore significantly shifted. In addition, several cross peaks in the region corresponding to serine or threonine residues displayed either reduction of intensity or significant chemical shift changes in the presence of liposomes. These observations suggest that the dynamic properties of the disordered region of NS4 are significantly affected upon liposome interaction. Particularly, the region encompassing residues N138 to G159 would become less mobile. Finally, a number of peaks exhibited splitting, suggesting the presence of two NS4 subpopulations in the sample, that are presumably the liposome-associated and liposome-independent NS4 fractions found in membrane flotation assays.

We then turned to cryo-EM to image the NS4:liposome mixture. A control liposome sample without NS4 showed separate small unilamellar vesicles with a round shape and a 100 to 300 nm diameter (Fig. 3*C*, left). In stark contrast, the NMR sample with NS4 showed only heavily clustered liposomes on grids, without any isolated liposomes (Fig. 3*C*, right).

Taken together, these results indicate that recombinant NS4 oligomerizes in the presence of liposomes into both membrane-bound and membrane-independent multimers and with local structuring in the region of residues N138-G159. Furthermore, the liposome-associated fraction of NS4 not only binds to liposomes, but seems to bridge them together.

### Distinct NS4 assemblies bridge synthetic liposomes

**Figure 4:**
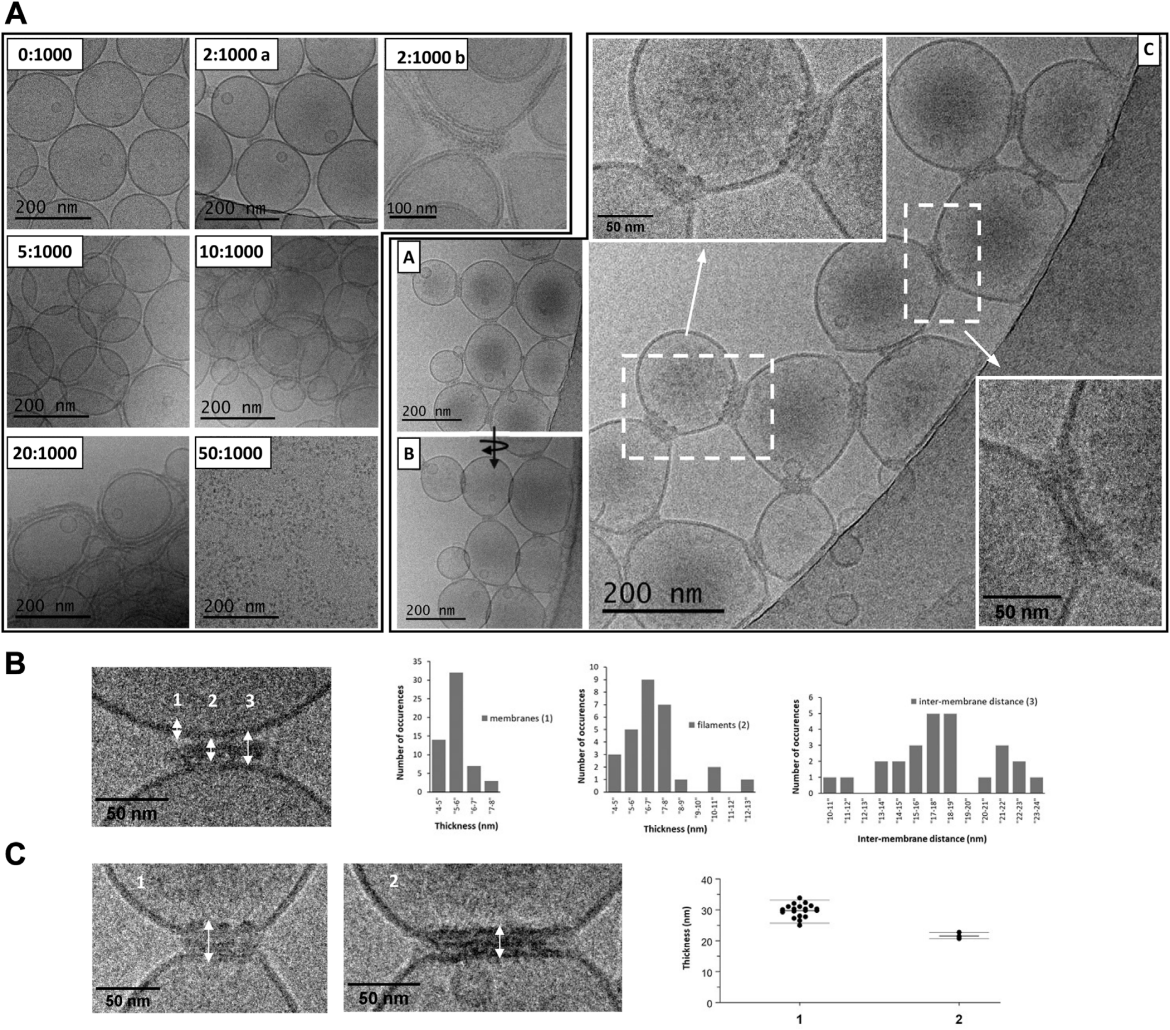
**Imaging of NS4-clustered liposomes and associated NS4 assemblies.** DDM-solubilized NS4 was incubated with PC-PS liposomes at multiple ratios and imaged by cryo-EM. *A*, *left*, representative images at the edges of liposome clusters for NS4:lipids ratios from 0:1000 (only NS4 buffer) to 20:1000 are shown. For 50:1000 liposome, clusters could not be included in a thin ice layer, showing on grids only objects that seem to be NS4 oligomers. 2:1000 b is an image from a separate experiment on a higher end microscope. *Right*, images from the grid indicated in *red* on Fig. S4*B*, at a ratio of 20:1000 NS4:lipids mol:mol (NS4 54 μM; lipids 2.6 mM). *B*, is the same location as (*A*) after a 30° tilt of the sample (indicated by an *arrow*). *C*, is a zoom on another region with the insets detailing the zipper-like structures bridging liposomes. *B* and *C*, quantification of intervening material between pairs of liposomes. See Experimental procedures and Fig. S4 for methodology. *B*, for 2:1000 micrographs, we measured membrane thickness as a control, filament thickness, and intermembrane distance. *C*, quantification of zippers and zipper-like objects (1 and 2, respectively) between bridged liposomes visible on the 20:1000 grid of Figure 4*A*, *right*. We measured thickness of the two distinct objects. DDM, n-dodecyl-β-D-maltoside; NS, nonstructural; PC, phosphatidylcholine; PS, phosphatidylserine.

In order to better understand NS4 behavior in the presence of liposomes, we next sought to get more precise cryo-EM views of these NS4 proteoliposomes. Apart from the usual workflow in cryo-EM of varying blotting time and force after deposition of the sample on grids and prior to plunge-freezing, we systematically explored combinations of concentrations of NS4 and lipids along 5 protein:lipids ratios (2:1000, 5:1000, 10:1000, 20:1000, and 50:1000 mol:mol). Altogether we imaged 17 NS4:lipids mixtures with NS4 concentration ranging from 4.3 μM to 90 μM and lipids from 1 mM to 6.2 mM (Fig. S3*A*). The overall results are summarized in the diagram and low magnification images of Fig. S3*B*: We found that exposure to NS4 leads to liposome clustering in all conditions. At higher concentrations of NS4 and lipids (*e.g.*, [NS4] > 50 μM and/or [lipids] > 3 mM), the liposome clusters induced by NS4 were too large to be caught into a vitreous ice layer thin enough to allow detailed high-magnification imaging by cryo-EM (“3D cluster” zone in the diagram of Fig. S3*B*). At lower NS4 concentration and lower NS4:lipids ratio, we got thinner clusters of liposomes that could be imaged more easily, particularly at their periphery where single layers of liposomes could be readily found (“2D cluster” zone). Higher magnification images (Fig. 4*A*) showed that, at a ratio of 2:1000, these clusters of liposomes, still round-shaped, showed stretches of intervening filamentous material between them (Fig. 4*A* and 2:1000 a). Grayscale profiles crossing this material and the two membranes on either side showed three regions at liposome contacts (Fig. S4*A*), with a lighter region between material and membranes. We thus measured three values at these contact sites: the thickness of the intervening material, the intermembrane distance and as a reference the thickness of the membranes themselves (Figs. S4*A* and 4*B*). The membranes’ thickness shows its expected distribution with a single narrow peak at 5 to 6 nm. In contrast, the thickness of the material shows a broad and multimodal distribution, with a broad peak around ∼7 nm but with other values extending to >12 nm. Similarly, the distribution of intermembrane distances is very broad and shows a major mode at ∼18 nm but also two others at ∼22 nm and ∼11 nm. A 2:1000 sample imaged on a higher-end microscope allowed a more precise view of this material (Fig. 4*A* and 2:1000 b): It is organized as bundles of filaments between pairs of apposed bilayers that do not show any visible strain. The marked heterogeneity in thickness could thus be due to varying numbers of filaments. Bridging material can still be distinguished at higher NS4:lipid ratios (particularly 5:1000) but at higher ratios the 3D nature of the clusters almost precludes clearly imaging contacts. Still we got a grid at ratio 20:1000 at lower NS4 and lipids concentrations with regions of thin ice where we could find 2D clusters of liposomes (Fig. S3*B*, red square; Fig. 4, *A–C*). These liposomes are bridged by structures (Fig. 4*A*, C, insets) distinctly different from the filamentous material observed at lower NS4:lipid ratios (Fig. 4*A* and 2:1000 b). Unlike the filaments, these structures span two tightly apposed membranes, although tilting the grid (Fig. 4*A*, B) showed that the structures do not seem to actually cross the bilayers and the membrane themselves do not seem to have merged or interdigitated. In pairs of bridged liposomes, the apposed membranes look straightened, making the liposomes more angular and giving the connecting structures the appearance of zippers. Because of these features, we could only measure one dimension across the apposed membranes, namely the thickness of these “zippers” (Figs. S4*B* and 4*C*). We found two distinct appearances, a majority of thicker zippers with apparent bulges beyond the membranes (Fig. 4*C*, left) and a minority of apparently thinner objects (Fig. 4*C*, middle). We cannot say whether the thinner zippers are actually different membrane-bridging assemblies or whether they are the same assemblies differently tilted on the grid. At any rate, both kinds of objects show narrow distributions of thickness, at 30 ± 2.1 nm and 22 ± 0.86 nm, respectively.

Finally, it may be noteworthy that in all 3D clusters obtained at 10:1000 and 20:1000, the liposomes that can be imaged at the edges look deformed (Fig. 4*A*, 10:1000 and 20:1000), although we cannot say whether it comes from the higher protein:lipid ratios in proteoliposomes (as established Fig. 3*A*, middle) or from the physical strain of depositing and blotting large 3D clusters of liposomes. We could not obtain cryo-EM images of liposomes at 50:1000, though negative staining EM showed clusters in the sample along with separate proteinaceous material (Fig. S5). These particles are readily imaged by cryo-EM at this protein ratio (Fig. 4*A*, 50:1000) and may be the liposome-independent NS4 multimers we found in membrane flotation assays (Fig. 3*A*).

## Discussion

Intracellular replication of (+)RNA viruses is associated with membrane alterations that can be extensive. This is the case for noroviruses, for which clusters of vesicles harboring the viral replication complex are induced upon infection, as best characterized with the genogroup V, murine viruses (7, 22). It has grown clear that the drivers of these large membrane rearrangements are the first three nonstructural proteins encoded in the norovirus replication polyprotein ORF1: NS1-2, NS3, and NS4 (8, 13). In norovirus replication, besides the fully processed form, NS4 is present as the N terminus of several transiently stable processing intermediates subsequent to the rapid cleavage of NS1-2 and NS3 (16, 17, 23). These intermediates are very effective ways of recruiting the critical viral factors NS5/VPg, NS6/protease, and NS7/polymerase to the membranous replication compartment. NS4, one of the most variable norovirus proteins, is also called for human noroviruses p22 or p20, or 3A-like in reference to the 3A protein in the replication polyprotein of picornaviruses (13). Like NS4, 3A is not only involved in intracellular alterations: It is also the N terminus of a processing intermediate including the picornaviral VPg, also the protein primer for positive-sense viral RNA synthesis. Thus, both 3A and NS4 are the recruiters of this essential factor to the replication complex. We previously noted similarities also in the general structural organization of NS4 to the HCV NS5A, despite the much larger size of the latter (some 450 residues) (8). Our AlphaFold models of NS4 (Fig. 2*A*) confirm this general likeness as a protein with a folded globular domain, an amphipathic helix and an intrinsically disordered region. The atomic level accuracy of AlphaFold models shows that NS4 is not homologous either to NS5A or to 3A. Indeed, the partial NMR structure of poliovirus 3A (residues 1–59 of 87) shows that the N terminus of 3A forms a dimer, with each monomer comprising an intrinsically disordered region followed by a pair of helices (24), a minimal fold unrelated to NS4’s (Fig. S6). Still, AlphaFold modeling of the full-length 3A dimer shows that its hydrophobic C terminus would adopt a long helical structure, completing a general organization strikingly similar to NS4’s (Fig. S6). We thus have here what seems to be a case of convergent evolution in noroviruses and picornaviruses. Together with hepaciviruses, this may point to a general suitability of this organization as a globular domain, a membrane-interacting helix and an intrinsically disordered region for the main membrane alteration protein of (+)RNA, DMV-type viruses.

Both 3A and NS5A form dimers, and NS5A’s membrane-remodeling functions are intimately linked to its self-assembling into higher order multimers. Indeed, a major mode of action of so-called “NS5A inhibitors,” the most potent antiviral drugs ever designed, lies in preventing this higher order self-association (25). Still our attempts at modeling oligomers of NS4 with AlphaFold were unsuccessful, unlike our previous success with the hepatitis E virus pORF1 N-terminal domain that forms a membrane pore (26) or our present success with the poliovirus 3A dimer (Fig. S6). This may point to the difficulty in modeling associated with a protein capable of forming several alternate assemblies (27), as we show here for NS4 (see below). But it may also be due to the fact that some protein–protein interactions do not seem to be conserved among Caliciviridae, raising contradictory coevolutionary signals in the multiple sequence alignments that AlphaFold uses as a guide. For instance, while a clear self-interaction of NS4 was found for murine norovirus (23), such an interaction was not observed for its counterpart in Feline Calicivirus (28). We show that human GII.4 NS4 does self-assemble onto membranes (Fig. 3*A*), and provide further data about NS4 structure by NMR spectroscopy. The NMR data provide a correction to the AlphaFold model by showing that in purified NS4, the most flexible part seems to be an intervening 20-residue region comprising the C terminus of the predicted amphipathic helix (N138-G159), rather than the extreme C terminus of NS4. Our NMR data further indicate major changes in this intervening region in the presence of liposomes. One conclusion is that self-assembly of NS4 involves structuring (or at least reduced mobility) of the residues at the C terminus of the amphipathic helix. Another is that at least two distinct conformations of the intervening region are formed, in accordance with several types of NS4 assemblies coexisting in the presence of membranes. Indeed, we find an unexpected fraction of NS4 oligomers that do not remain associated with membranes. Their status is puzzling. They may be involved in some other function not directly related to the membranous replication compartment formation. For instance, human (GI.1) norovirus NS4 was reported to interfere with ER/Golgi trafficking through a conserved ER export signal (29).

In norovirus replication, the fully processed NS4 seems to be the main driver of membrane rearrangements. Thus, individual expression of NS4 (but not NS1-2 or NS3) from human GII.4 norovirus is sufficient to induce the formation of vesicles, including SMV and DMV, similar to those found in murine norovirus infection (8). However, NS4 also interacts with cellular factors, for instance the above-mentioned interference with ER/Golgi trafficking (29). It remains unclear which of an intrinsic NS4 membrane activity or interactions with cellular factors is most important in the NS4-induced vesicular system phenotype. Here, we used cryo-EM to establish clear membrane activities of recombinant human (GII.4) NS4 in the absence of any other factor. By itself, NS4 is sufficient to cluster synthetic liposomes together (Fig. 3*C*, 4, and S3). This property of membrane remodeling by spontaneous self-assembly is again reminiscent of HCV NS5A. Remarkably, even in this most basic system (NS4 and two lipids), NS4 is shown here to form at least three different assemblies: the aforementioned membrane-independent oligomers, bundles of filaments between membranes, and membrane zippers. Membrane-independent oligomers are formed even at lower NS4:lipids ratios (2:1000, Fig. 3*A*), although due to their smaller size they become readily detectable by cryo-EM only at 50:1000, where they are more abundant and liposomes are so bunched together that they are no longer included in thin ice (Fig. 4*A*). At the lower ratios the oligomers coexist with the bundles of filaments. Although the bundles cluster liposomes together (Fig. 4*A*), they seem only loosely connected to liposomes, with no apparent change of the latter (Fig. 4*A* and 2:1000) and varying bundle thickness and intermembrane distance (Fig. 4*B*). This is in stark contrast to the zippers, which clearly bridge pairs of membranes seemingly apposed together at defined distances by structures that look to extend from one membrane to the other (Fig. 4, *A* and *C*). This visual impression of homogeneity is borne out by quantification of the apparent thicknesses of the zipper-like objects (Fig. 4*C*). This kind of structures is well suited to the formation or stabilization of double-membrane systems. Fully processed NS4 accumulates during the course of norovirus infection. We may thus speculate that our separate experiments increasing NS4:lipids ratios may mimic features of a time course of membrane rearrangements. In this scenario, the bundles would represent early assembly forms of NS4, while the zippers would appear and probably coexist with bundles at later time points. In accordance with this, the membranes in our simple NS4:liposomes system become more convoluted as the NS4:lipids ratio is raised, showing features of double-membrane vesicles at 20:1000, where we could also find the zippers. Small unilamellar liposome preparations are known to be in a metastable state. For instance, dehydration of the water film in the holes of the grids prior to plunge freezing can induce membrane invaginations by itself (30). It is thus noteworthy that there is no discernible direct liposome deformation by NS4 in our experiments, except a straightening around the zippers. We are brought to a model in which NS4, while not a membrane-deforming protein on its own, would adopt several distinct membrane-bridging forms. These forms, in conjunction with other viral and cellular factors likely contribute to the formation of the various membrane alterations observed during norovirus replication.

## Experimental procedures

### Cloning and purification of NS4

The coding region of full-length NS4 from the GII.4/Helensburgh/NSW295E/2010/AU isolate of the NO 2009 GII.4 strain (GenBank accession n° JQ613573) was PCR-amplified from the pTM-ORF1_NO plasmid (8) and inserted into the pET16b vector between NcoI and XhoI restriction sites. The resulting protein, from residue Gly697 to residue Glu875 (polyprotein numbering), contained an extra initiating methionine followed by a noncleavable 6His-tag in its N-terminal extremity (Fig. S1). The plasmid was amplified in NEB Turbo thermo competent cells (New England Biolabs), purified using a Nucleo Spin Plasmid kit (Macherey-Nagel) and verified by sequencing (Eurofins Genomics).

BL21(DE3) cells transformed with plasmid encoding 6His-NS4 were grown at 37 °C in LB medium supplemented with ampicillin. Protein expression was induced with 0.3 mM IPTG for 5 h at 37 °C. Cells were harvested by centrifugation, resuspended in lysis buffer (50 mM Tris–HCl, 300 mM NaCl, 20 mM imidazole, 0.5% Triton X-100, 5 mM beta-mercaptoethanol, lysozyme 1 mg/ml, pH 7.4) supplemented by home-made benzonase and EDTA-free protease inhibitor cocktail (Roche), and incubated 1 h at 4 °C (80 ml of lysis buffer for 5 L culture). Cells were disrupted by sonication and the lysate was centrifuged at 20,000*g* for 30 min at 4 °C. The supernatant was purified by Ni-affinity chromatography using a 5 ml HisTrap HP column (Cytiva). The column was equilibrated with buffer A (50 mM Tris–HCl, 300 mM NaCl, 20 mM imidazole, 0.02% Triton X-100, 5 mM beta-mercaptoethanol, pH 7.4), and the protein was eluted with a linear gradient of buffer B (50 mM Tris–HCl, 300 mM NaCl, 500 mM imidazole, 0.02% Triton X-100, 1 mM DTT, pH 7.4). Fractions containing NS4 were pooled and further purified by cation exchange chromatography. For that, the pool was diluted two times, the concentrations of DTT and Triton were adjusted to 1 mM and 0.1%, respectively, and EDTA-free protease inhibitor cocktail (Roche) was added. Finally, the pH of the solution was adjusted to 6.5 with concentrated MES buffer. The resulting NS4 pool was loaded on a 5 ml HiTrap SP FF column (Cytiva) pre-equilibrated with buffer C (25 mM Tris–HCl, 150 mM NaCl, 0.1% Triton X-100, 1 mM DTT, pH 6.5). Protein was eluted with a linear gradient of buffer D (25 mM Tris–HCl, 1 M NaCl, 0.1% Triton X-100, 1 mM DTT, pH 6.5). In order to concentrate the protein without concentrating the detergent, the fractions containing NS4 were pooled and incubated 3 h at 4 °C under gentle agitation with 500 μl of Ni-NTA Agarose resin (QIAGEN) pre-equilibrated with a buffer composed of 50% buffer C and 50% buffer D. Triton was replaced by DDM by incubating the resin 30 min at 4 °C under agitation with washing buffer (10 mM MES, 150 mM NaCl, 1 mM DTT, pH 6.5) containing 0.05% Triton X-100 and 0.04% DDM first, then 0.17% DDM, then 0.04% DDM. Protein was eluted with buffer H (10 mM MES, 150 mM NaCl, 0.04% DDM, 1 mM DTT, 500 mM imidazole, pH 6.5). Finally protein was dialyzed at 4 °C with a 10 kDa cutoff dialysis membrane against buffer I (10 mM MES, 150 mM NaCl, 0.5 mM tris(2-carboxyethyl)phosphine (TCEP), pH 6.5), first 1 h then overnight. Concentration was estimated from the extinction coefficient ε^280^ = 14,440 M^-1^ cm^-1^ calculated from the sequence. Protein was used within 2 weeks or stored at −80 °C.

The ^15^N-labeled protein was expressed in 2 L of minimal (M9) medium containing 1 g/L ^15^NH_4_Cl and 4 g/L [^12^C] glucose. The ^15^N-labeled protein was then purified following the same protocol as the unlabeled one.

### Generation of anti-NS4 mAb

For specific detection of recombinant NS4, a monoclonal anti-NS4 antibody was generated with the support of the Core Facility Antibodies of the German Cancer Research Center (DKFZ), according to the principles of Köhler and Milstein's hybridoma technology (31). In brief, BALB/c mouse immunization was performed with purified 6His-NS4. Anti-NS4 antibody–producing B-lymphocytes isolated from positively reacted mice were fused with Sp2/0 murine myeloma cells (RRID: CVCL_2199) to produce hybridoma cell clones. Two clones were selected to produce two distinct mAbs. Both antibodies were purified from their supernatant using two distinct 5 ml HiTrap protein A columns. Loading and washing were done in 50 mM phosphate buffer at pH 7.5 for five column volumes. Elution was performed in 100 mM glycine buffer pH 2.5. The eluted fractions were combined and first dialyzed overnight at 4 °C in 1 L of a 50 mM potassium phosphate buffer at pH 7.5, and then another 2 h in a new freshly made buffer.

### Size-exclusion chromatography

Alternatively to dialysis as the last step of purification process, NS4 could be applied on a Superdex 200 Increase 10/300 GL column (Cytiva) and eluted at a flow rate of 0.5 ml/min in 10 mM MES, 150 mM NaCl, 0.04% DDM, pH 6.5 buffer. The column was calibrated with molecular markers (Cytiva): Blue Dextran 2000 (∼2000 kDa), ferritin (440 kDa), aldolase (158 kDa), and ovalbumin (43 kDa).

Analytical size-exclusion chromatography experiments were performed on a Superdex 200 5/150 column (Cytiva) equilibrated in 10 mM MES, 150 mM NaCl, and 0.04% DDM, pH 6.5. Samples containing decreasing concentration of NS4 (ranging from 2.7 to 0.3 mg/ml) were prepared in a final volume of 90 μl, centrifuged (10 min, 20,000*g* at 4 °C), and loaded onto the column. Elution was performed in the same buffer at a constant flow rate (0.3 ml/min). Molecular weight markers (ferritin (440 kDa), aldolase (158 kDa), ovalbumin (44 kDa), carbonic anhydrase (29 kDa), and ribonuclease A (14 kDa)) were analyzed in the same conditions. The peaks were integrated with the Unicorn software provided with the AKTA FPLC (Cytiva) we used for this experiment.

### Wheat-germ cell-free expression of ORF1

ORF1 from two other GII.4 strains, a Sydney 2012 variant and a Den Haag 2006b variant (GenBank accession n° JX459908 and AB447456, respectively), WT or carrying the C1147A mutation, were expressed by *in vitro* translation as previously described (15). Full-length ORF1 or its maturation products were detected by Western blot using anti-NS4 antibody.

### Biochemical characterization

For both SDS-PAGE conditions, we used a standard homemade Laemmli buffer composed of 250 mM Tris–HCl pH6.8, 8% SDS, 40% glycerol, 0.2% bromophenol blue, with or without 20% β-mercaptoethanol for the reducing or nonreducing condition, respectively.

For Western blotting, proteins were denatured and reduced in Laemmli buffer and loaded onto a 14% SDS-PAGE. After migration, proteins were transferred to a nitrocellulose membrane (Amersham Protran 0.45 NC, Cytiva). Immuno-detection was performed by incubating the membranes with the monoclonal anti-NS4 antibody (1:100,000 dilution factor) for 1 h. Primary antibodies were detected using anti-mouse secondary antibodies coupled to horseradish peroxidase-coupled secondary antibodies (Sigma-Aldrich) and revealed by enhanced chemiluminescent substrate.

Mass spectrometry identification was performed on an SDS-PAGE of a purified NS4 sample. The single band at 21 kDa was cut out and trypsin-digested prior to being analyzed by liquid chromatography-mass spectrometry (Nano Elute and TimsTOF Pro, Bruker).

### Molecular modeling

Structure of NS4 from the NO 2009 variant of huNoV GII.4 (GenBank accession n°JQ613573, residues Gly697 to Glu875) was modeled using our inhouse ColabFold (v. 1.5.2) pipeline of AlphaFold (v. 2.3). 15 models were generated and ranked. PYMOL (http://citebay.com/how-to-cite/pymol/) was used to display two of the best-scored five on Figure 2*A*.

### Liposome preparation

Lipids (egg PC and brain PS, Avanti Polar lipids) were resuspended in chloroform and mixed in a round-bottom flask to obtain 89% egg PC (0.89 mM) and 10% brain PS (0.1 mM). The fluorescent dye Laurdan was also added (final concentration 1% (0.01 mM)). The solvant was evaporated in a Rotavapor and the resulting lipid films were resuspended with 10 ml of milliQ H_2_O for 2 h at 22 °C, with additional 5 ml to rinse the flask. The lipid films were then aliquoted into ten 2 ml Eppendorf tubes and lyophilized overnight before being filled with argon and stored at −20 °C. Lipid films were hydrated under gentle agitation in 10 mM MES, 150 mM NaCl, pH 6.5 buffer to reach a concentration of 6.5 mM. Lipids were then solubilized with Anapoe-X-100 (Anatrace) with a detergent/lipid mass ratio of 2.5 for 15 min at room temperature under gentle stirring. Liposomes were formed by adding biobeads (biobead/detergent mass ratio of 20) and incubating the mixture at 20 °C with stirring for 2 h. The operation was repeated two times, with 1 h incubation period. Liposomes were collected with a long and thin tip and stored at 4 °C for a few days. Homogeneity of the suspension and size distribution of liposomes were controlled by dynamic light scattering using a diluted sample (0.1 mM).

### Liposome flotation assay

PC:PS:Laurdan liposomes (89/10/1%) were incubated in Beckman polycarbonate centrifuge tubes with 6His-NS4. Three samples were prepared with following composition: NS4 alone and two samples at a 2:1000 and 20:1000 mol:mol NS4:lipids ratio incubated for 10 min before centrifugation (1.7 and 17 μM NS4 and 850 μM lipids). The mixture (200 μl) was incubated at room temperature and mixed with 10 mM MES, 150 mM NaCl, 0.01% Triton X-100, and pH 6.5 buffer containing 40% sucrose (200 μl). The suspension was overlaid with three layers of the same buffer (400 μl each) containing sucrose at 15%, 10% and 5% successively and finally 200 μl of buffer without sucrose. After centrifugation at 258,000*g* in a TLS-55 swinging rotor (Beckman) for 2 h at 15 °C, eight fractions of 225 μl were collected from top to bottom. All fractions were analyzed by fluorescence to detect Laurdan-containing liposomes and by Western blot using anti-NS4 antibody to detect the protein.

### Cryo-EM experiments and measurements of dimensions of assemblies

Quantifoil R3.5/1200-mesh grids were used in cryo-EM experiments (except the single image of Fig. 4*A* and 2:1000 b, for which a lacey grid was used). PC:PS:Laurdan liposomes (in buffer 10 mM MES, 150 mM NaCl, and pH 6.5 supplemented with 0.5 mM TCEP) were incubated at 20 °C with 6His-NS4 (in buffer 10 mM MES, 150 mM NaCl, 0.5 mM TCEP, 0.04% DDM, and pH 6.5) at different concentrations and ratios depending on the experimental conditions (Fig. S3*A*). Five microliters of sample were applied to glow-discharged grids before blotting and plunge-freezing in liquid ethane using a Vitrobot Mark IV (FEI) at 100% humidity (except Fig. 4*A* and 2:1000 b, where plunge-freezing was performed with an inhouse guillotine). Blotting time and force were adjusted on a per sample basis. Images were acquired with a Tecnai Spirit 120 kV LaB6 FEI microscope operated at 120 kV equipped with a K2 Base camera (Gatan) (except Fig. 4*A* and 2:1000 b, where the image was acquired with a Glacios 200 kV FEG microscope equipped with a Falcon 4 camera (ThermoFisher Scientific)).

Dimensions of assemblies were measured using FIJI (https://fiji.sc/) and ImageJ (https://imagej.net/ij/) softwares for the filamentous material (Figs. S4*A* and 4*B*) and the zipper-like assemblies (Figs. S4*B* and 4*C*). We oriented the image so that the object to measure was in a vertical position, we framed the object, we plotted the horizontal, vertically averaged profile, and then exported the data (gray value and distance) to Excel in order to plot the graph and determine distances. For filaments (Fig. S4*A*), we measured bundles of filament thickness, membrane thickness, and intermembrane distance from 26 objects selected from 8 micrographs. For zippers, we measured the overall thickness of the connection between two apposed membranes from 25 objects selected from 10 micrographs.

### NMR experiments

NMR experiments were performed on a 700 MHz Bruker AVANCE NEO spectrometer equipped with a triple resonance cryogenic probe. To follow the NMR behavior of the DDM-solubilized NS4 protein in interaction with liposomes, 2D ^1^H-^15^N SOFAST-HMQC experiments were recorded (32) at 308 K. NMR spectra were collected at protein concentrations of either 150 μM for NS4 alone or 56 μM for NS4 with 4.7 mM liposomes (final protein:lipids ratio of 12:1000 mol:mol), using 3 mm diameter NMR tubes. For these experiments, the buffer consisted of 10 mM MES, 150 mM NaCl, 0.04% DDM, and 0.5 mM TCEP at pH 6.5.

## Data availability

All data are contained in the article and the supporting information.

## Supporting information

This article contains supporting information (24, 33, 34).

## Conflict of interest

The authors declare that they have no conflicts of interest with the contents of this article.
