# Supplementary material for: Nonstructural protein 4 of human norovirus self-assembles into various membrane-bridging multimers
Source: J Biol Chem. 2024 Aug 28;300(9):107724. doi: 10.1016/j.jbc.2024.107724 (PMC11439542; doi:10.1016/j.jbc.2024.107724)
Supplement: Supporting information [file mmc1.pdf]

Nonstructural protein 4 of human norovirus self-assembles into various membrane-bridging multimers

Supporting Information

Adrien Royet, Rémi Ruedas, Laetitia Gargowitsch, Virginie Gervais, Johann Habersetzer, Laura Pieri, Malika Ouldali, Maïté Paternostre, Ilse Hofmann, Thibault Tubiana, Sonia Fieulaine, Stéphane Bressanelli

Supplementary References

1. Deléage, G. (2017) ALIGNSEC: viewing protein secondary structure predictions within large multiple sequence alignments. *Bioinformatics*. **33**, 3991–3992
2. Robert, X., and Gouet, P. (2014) Deciphering key features in protein structures with the new ENDscript server. *Nucleic Acids Res*. **42**, W320-324
3. Strauss, D. M., Glustrom, L. W., and Wuttke, D. S. (2003) Towards an understanding of the poliovirus replication complex: the solution structure of the soluble domain of the poliovirus 3A protein. *J Mol Biol*. **330**, 225–234

Supplementary Figure Legends

Supplementary Figure S1: Sequence alignment of NS4 belonging to three GII.4 variants.

The sequence of recombinant New Orleans (NO) 2009 NS4, fused to a 6His-tag at its N-terminal extremity (identified as rec_NewOrleans on the figure), was aligned with NS4 from Sydney 2012 and Den Haag 2006 variants. Numbering corresponds to the NO sequence, in the recombinant protein and in the ORF1 polyprotein (in brackets). Alignment was performed by CLUSTALW (1) and displayed with ESPript3 (2). White characters in red boxes indicate identity, and red characters in white boxes indicate homologous residues (non homologous residues are colored in black). The last three cysteines and Gly159 are highlighted in blue and yellow, respectively. Secondary structures of NO NS4 AlphaFold model rank 1 are indicated on top. Black stars below the alignment indicate the length of the synthetic peptide we used in this study (Sydney sequence).

Supplementary Figure 2: Purification of recombinant NS4

A, SDS-PAGE analysis of Ni-NTA affinity chromatography performed on cell lysate

B, SDS-PAGE analysis of cation exchange chromatography performed on pooled fractions (16 to 31) from previous Ni-NTA affinity chromatography

C, SDS-PAGE analysis of Ni-NTA affinity chromatography (batch) performed on pooled fractions (9 to 31) from previous cation exchange chromatography

D, Size-Exclusion chromatography performed on fraction E2 from previous Ni-NTA affinity chromatography and its associated SDS-PAGE analysis.

Supplementary Figure 3: Imaging of NS4-clustered liposomes and associated NS4 assemblies

DDM-solubilized NS4 was incubated with PC-PS liposomes at multiple ratios (concentrations ranging from 1.04 mM to 6.2 mM for the lipids; from 4.3 µM to 90 µM for NS4) and imaged by cryo-EM.

A, The table lists all the conditions we tested, at various concentrations and ratios. For each sample, several blotting conditions were usually tried to find a grid suitable for imaging.

B, The diagram summarizes unique conditions tested. The lower magnification images around the diagram at the ends of arrows are representative of the typical aspects of the grids in the given condition upon observation. The dotted arrow indicates another condition leading to similar observations. For unique conditions for which distinctly different aspects were obtained in different experiments, two images are given (see ***A*** for the numbers of repeats). One such condition in which we could image NS4 membrane zippers is signaled with a red arrow and a red square. '2D cluster' locates the zone of the diagram where clustered liposomes can still be easily caught and imaged in a thin ice layer. '3D clusters' indicates a part of the diagram where liposomes are most often too clustered along the observation axis for imaging contacts between pairs of liposomes.

Supplementary Figure 4: Methodology for measuring the thickness of objects formed by NS4 in contact with membranes

Various objects were selected in FIJI/ImageJ software (indicated by a frame), and plotted in order to obtain an intensity (gray value) as a function of the distance. This allowed us to determine the thickness of filaments and membrane (**A**), inter-membrane distance (**A**), and zippers' thickness (**B**).

Supplementary Figure 5: Electron micrograph of negatively stained NS4:liposomes at 50:1000

PC-PS liposomes incubated with DDM-solubilized NS4 at a 50:1000 NS4:Lipids mol:mol ratio (1.04 mM Lipids ; 54 µM NS4) was imaged by negative-stain electron microscopy (1% uranyl acetate).

Supplementary Figure 6: Comparison of structural models of GII.4 NS4 and poliovirus 3A

Models are colored by chain.

A, rank 1 GII.4 NS4 AlphaFold model as in Fig. 2A.

B, NMR model of the dimeric N-terminal domain of 3A (Strauss et al., *J Mol Biol* 2003).

C, AlphaFold model of the full-length 3A (this work).

Supplementary Figure 1

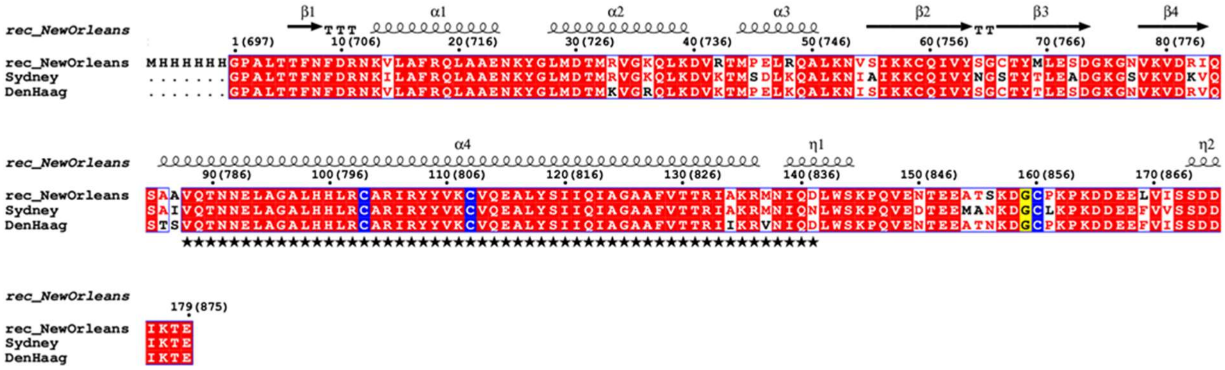

Supplementary Figure 2

A Ni-NTA affinity chromatography

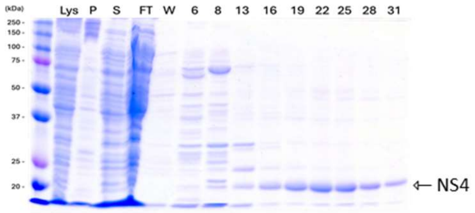

B Cation exchange chromatography

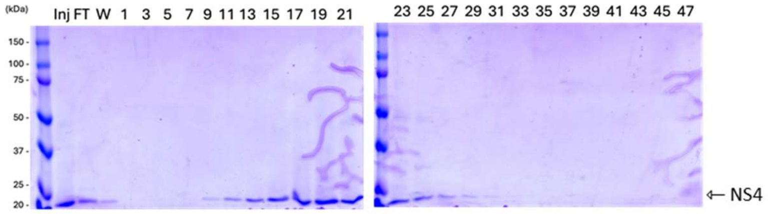

C Ni-NTA affinity chromatography

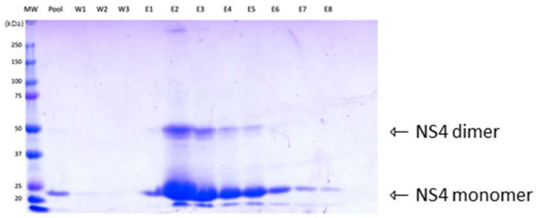

D Dialysis

Size exclusion chromatography

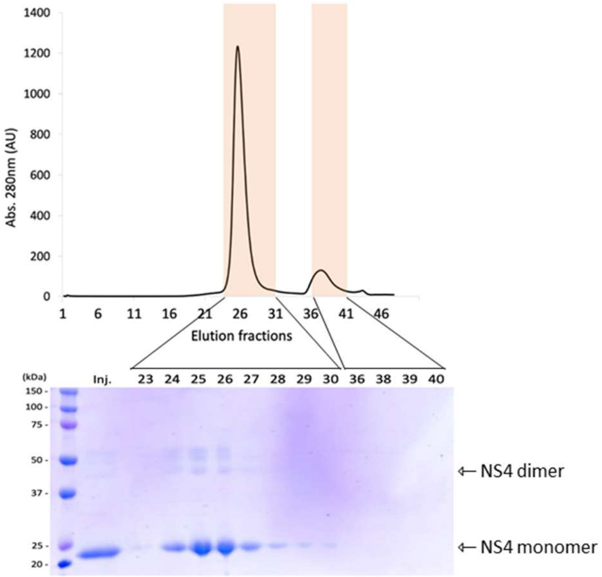

Supplementary Figure 2: Purification of recombinant NS4

A – SDS-PAGE analysis of Ni-NTA affinity chromatography performed on cell lysate

B – SDS-PAGE analysis of cation exchange chromatography performed on pooled fractions (16 to 31) from previous Ni-NTA affinity chromatography

C – SDS-PAGE analysis of Ni-NTA affinity chromatography (batch) performed on pooled fractions (9 to 31) from previous cation exchange chromatography

D – Size-Exclusion chromatography performed on fraction E2 from previous Ni-NTA affinity chromatography and its associated SDS-PAGE analysis.

Supplementary Figure 3

A

Ratio NS4:lipids (mol:mol)	NS4 (µM)	Lipids (mM)	Repeat
**2:1000**	4.3	2.2	1
	13.5	6.2	3
**5:1000**	16	3.05	1
	30	5.77	1
**10:1000**	30	2.89	1
	54	5.3	3
**20:1000**	54	2.6	5
	90	4.3	1
**50:1000**	54	1.04	1

B

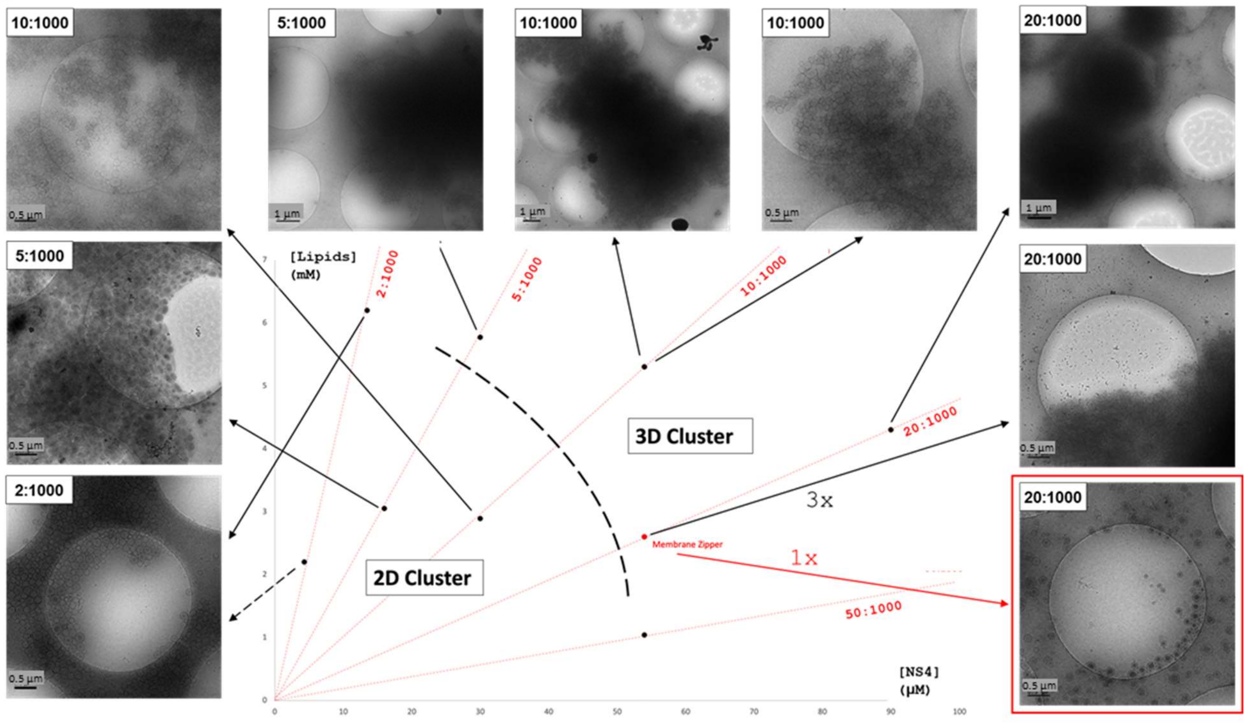

Supplementary Figure 4

A

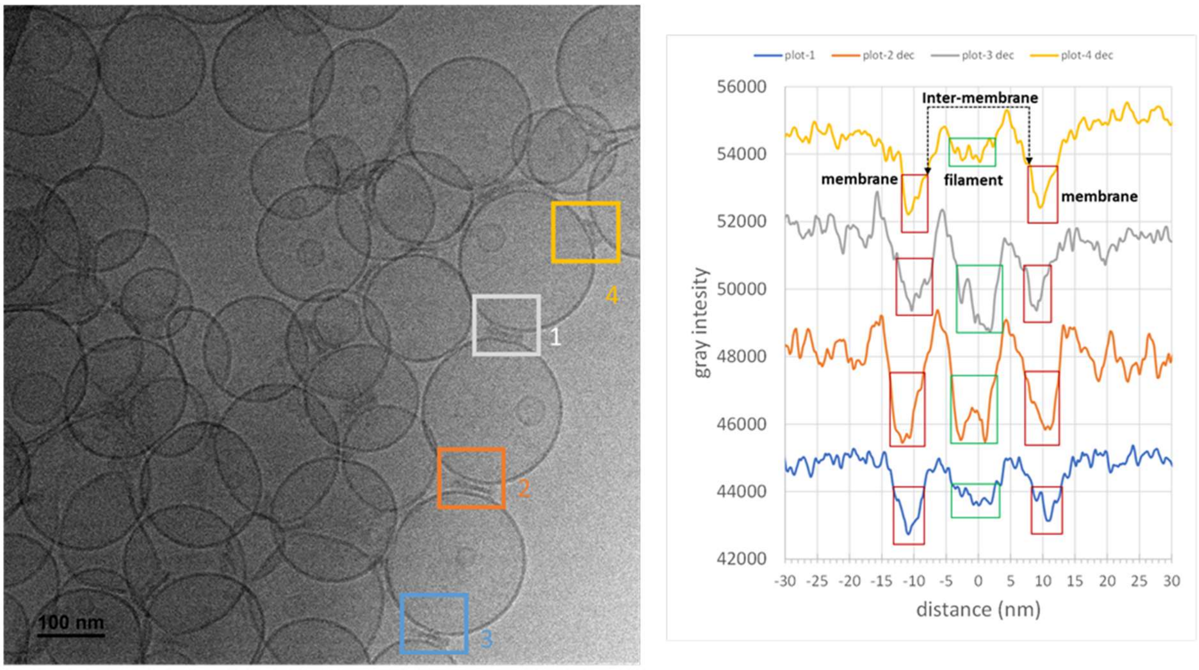

B

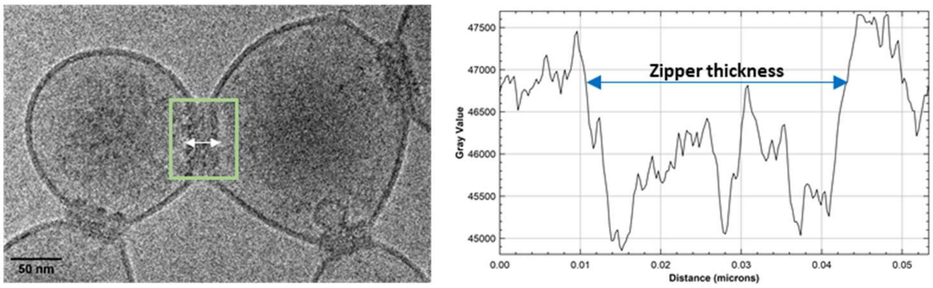

Supplementary Figure 5

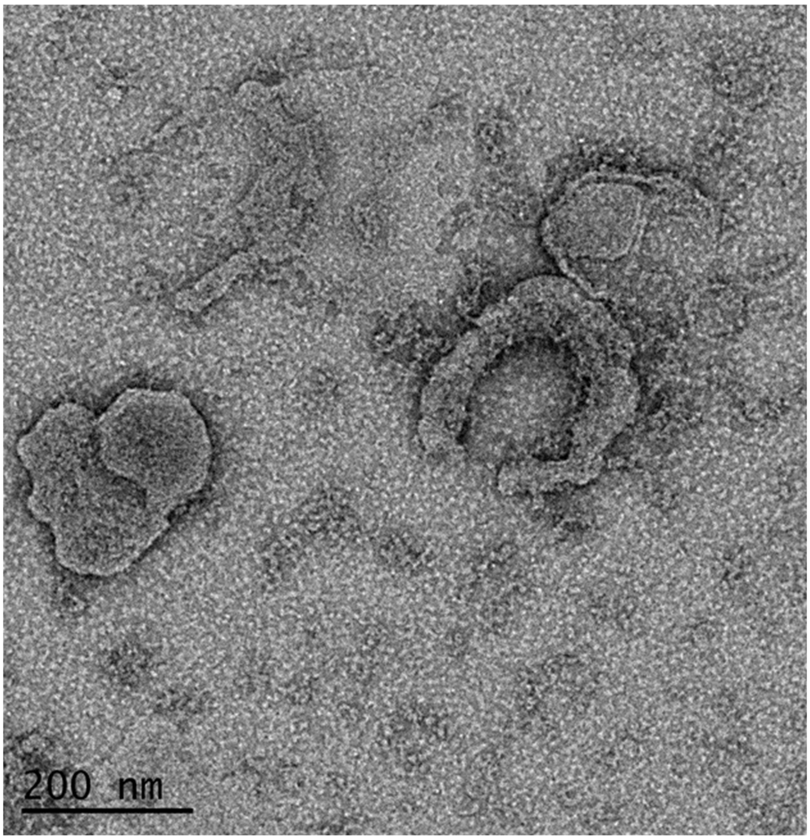

Supplementary Figure 6

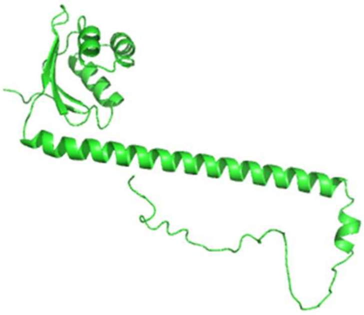

NS4 (1-179, AlphaFold)

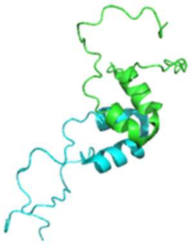

PV 3A dimer (1-59, PDB 1NG7)

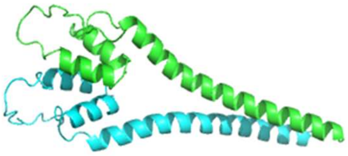

PV 3A dimer (1-87, AlphaFold)
